# Decompression syndrome (Caisson disease) in an Indian diver

**DOI:** 10.4103/0972-2327.70868

**Published:** 2010

**Authors:** Uday A. Phatak, Eric J. David, Pravin M. Kulkarni

**Affiliations:** Department of General Medicine, Yashodhan Hospital, Miraj, India

**Keywords:** Caisson’s disease, decompression syndrome

## Abstract

Acute decompression syndrome (Caisson’s disease) is an acute neurological emergency in divers. It is caused due to release of nitrogen gas bubbles that impinge the blood vessels of the spinal cord and brain and result in severe neurodeficit. There are very few case reports in Indian literature. There are multiple factors in the pathogenesis of Acute decompression syndrome (Caisson’s disease) such as health problems in divers (respiratory problems or congenital heart diseases like atrial septal defect, patent ductus arteriosus etc), speed of ascent from the depth and habits like smoking that render divers susceptible for such neurological emergency. Usually, immediate diagnosis of such a condition with MRI is not possible in hospitals in the Coastal border. Even though, MRI is performed, it has very low specificity and sensitivity. Facilities like hyperbaric oxygen treatment are virtually non-existent in these hospitals. Therefore, proper education of the divers and appropriate preventive measures in professional or recreational divers is recommended.

Though India has a coastline of 7500 km, not many cases of the decompression syndrome (DCS) or Caisson disease have been reported in Indian literature. The incidence of the DCS in the US is 1 per 5,000–10,000 dives per year, and the mortality is around 10%. There are two subtypes of DCS. Type I DCS or ‘the bends’ is characterized by vague symptoms like generalized weakness, body ache, abdominal pain, and vomiting, while type II DCS is associated with systemic dysfunction, presenting with cardiovascular involvement (‘the chokes’) or neurological features like paraplegia and bladder dysfunction.[1] We present a case of DCS in a young breath-holding diver who developed acute bilateral deafness and paraplegia.

## Case Report

NS, a 40-year-old male from the Konkan region of Maharashtra state, had been working with a bridge-construction firm for many years as a diver. His work involved periodically diving in the sea up to a depth of 20–30 meters to inspect bridge columns. He was a chronic smoker. He had no past history of deafness or neurological disease.

On 8^th^ April, 2008, he dived into the sea for inspection of the columns and returned to surface within a few seconds. While returning, he experienced severe pain in the back and neck region, weakness in both lower limbs, and marked muscle pain all over the body. He was shifted to a medical facility, where he was treated with nonsteroidal anti-inflammatory drugs and intravenous fluids. He continued to experience severe pain in the back and neck region and the extremities. His hearing was also affected and he faced difficulties in communication. In addition, he complained of abdominal pain, giddiness, and vomiting. He was shifted to our institution for management 10–12 h after the incident.

On admission, he complained of severe pain in the back, neck, and both lower limbs. He also had urinary retention. He had hypotension (80/60 mm Hg), tachycardia (140/min), tachypnea (30/min), and slight confusion. There were no rhonchi or crepitations. The heart sounds were normal. There was hypotonia in both lower limbs, with patchy sensory loss up to the level of the umbilicus, areflexia, and retention of urine. The cranial nerves were normal. Cognitive functions were also normal.

Investigations revealed hemoglobin 14.2 g/dl, white blood cell count 14,900 per mm^3^, platelet count 92,000 per mm^3^, blood sugar 126 mg/dl, blood urea 42 mg/dl, and serum creatinine 0.89 mg/dl. Serum electrolytes were normal. Serum creatine phosphokinase (CPK) was high (1192 U/l). Arterial blood gas analysis was normal. HIV test was nonreactive. Electrocardiogram and x-ray chest were normal. Audiometry showed bilateral sensorineural deafness. Nerve conduction velocity / electromyography (NCV/EMG) revealed radiculo-neuropathy, predominantly axonal neuropathy. Computed tomography (CT) scan of the chest and spinal cord were advised but, due to financial problems, both the tests could not be done on the day of admission. Acute DCS type II, with bilateral sensorineural deafness due to barotrauma, was diagnosed. He was given supportive treatment in the form of parenteral fluids, antibiotics, methylprednisolone, and oxygen. Within 48 h, the pain subsided. His general condition improved, but the paraparesis and deafness persisted. Magnetic resonance imaging (MRI) of the spinal cord done after 3 weeks was inconclusive.

Within 2–3 weeks, partial neurological improvement occurred and he could stand with crutches. Although he regained urinary control, hearing was possible only with the use of a hearing aid. Due to the persisting neurodeficit, he could not continue with his job. He is presently undergoing physiotherapy.

## Discussion

This patient had acute DCS, type II, characterized by acute radiculomyelopathy with bilateral sensorineural deafness, transient thrombocytopenia, and elevated CPK levels. He could not receive hyperbaric oxygen during the entire course of his illness.

Acute DCS has only rarely been reported in Indian literature. In 1964, Tripathy and colleagues reported a case of DCS in an Indian diver who developed acute recurrent paraplegia despite being treatment in a hyperbaric oxygen chamber.[4]

An association between thrombocytopenia and mortality has been established in animal studies, with thrombocytopenia being considered a marker of severity of the DCS. Platelets adhere to nitrogen bubbles in severe DCS, leading to the thrombocytopenia.[2] Raised level of CPK have been attributed to rhabdomyolysis.[3]

The nitrogen bubbles that form in the circulation during the phase of de-decompression are normally filtered out by the pulmonary capillaries. However, in the presence of anatomic defects such as an atrial septal defect or a patent foramen ovale, the risk of DCS increases significantly.[5]

The spectrum of neurodeficits following DCS is wide. It may range from cognitive dysfunction, cranial nerve lesions, and spinal cord dysfunction to complications due to barotrauma. The spinal cord may be damaged either due to disruption of the white matter or due to the formation of platelet microthrombi in the spinal circulation.[6]

Sensorineural deafness in acute DCS may be asymmetrical or symmetrical. However, unilateral deafness is seen more commonly than bilateral deafness. Klingmann et al. studied this problem in 46 patients and found that only one had bilateral deafness, the rest having only unilateral involvement. The other manifestations reported by the subjects were tinnitus and vertigo.[7]

Multiple factors are involved in the pathogenesis of the DCS. Divers with bronchial asthma, atrial septal defect, patent foramen ovale, or obesity are more prone to develop DCS. The depth of the dive below the sea surface, the temperature of the water, and the speed of ascent are considered as the main contributory factors for development of DCS. When divers ascend at a speed of 9–10 meters/min, they have minimal risk of developing DCS. If the ascent is faster (>19 meters/min), the risk of DCS is significantly higher.[8]

DCS is always a clinical diagnosis. The specificity and sensitivity of MRI for the detection of DCS is low. Radiological changes may be seen in the early stage of the disease but it has been reported that after 3 weeks significant regression would have occurred in these changes despite persistence of profound neurodeficit.[9]

Does late referral of DCS to a specialty hospital influence the long-term outcome? In a study of 140 patients with DCS, 44% of the patients had mental aberration, eventually all recovered. The median delay was 48 h. Complete recovery was seen in 87%. Oxygen therapy and administration of corticosteroids like methylprednisolone can be effective in the treatment of DCS when the hyperbaric oxygen chamber facility is unavailable.[10]

In brief, acute DCS in Indian divers has only rarely been reported. In this country there no formal training is given for recreational or professional divers regarding the precautionary measures to be taken for prevention of DCS. It is possible to avert such injuries with proper training and education. For example, divers should use ear-plugs for protection from barotrauma.
